# Genetic basis and identification of candidate genes for salt tolerance in rice by GWAS

**DOI:** 10.1038/s41598-020-66604-7

**Published:** 2020-06-19

**Authors:** Jie Yuan, Xueqiang Wang, Yan Zhao, Najeeb Ullah Khan, Zhiqiang Zhao, Yanhong Zhang, Xiaorong Wen, Fusen Tang, Fengbin Wang, Zichao Li

**Affiliations:** 1 State Key Laboratory of Agrobiotechnology / Beijing Key Laboratory of Crop Genetic Improvement, College of Agronomy and Biotechnology, China Agricultural University, Beijing, 100193 China; 2 Institute of Nuclear and Biological Technologies, Xinjiang Academy of Agricultural Sciences, Urumqi, 830091 China; 3 Rice Experiment Stations in WenSu, Xinjiang Academy of Agricultural Sciences, Aksu, 843000 China

**Keywords:** Genome informatics, Genome-wide association studies, Plant genetics

## Abstract

Soil salinity is a major factor affecting rice growth and productivity worldwide especially at seedling stage. Many genes for salt tolerance have been identified and applied to rice breeding, but the actual mechanism of salt tolerance remains unclear. In this study, seedlings of 664 cultivated rice varieties from the 3000 Rice Genome Project (3K-RG) were cultivated by hydroponic culture with 0.9% salt solution for trait identification. A genome-wide association study (GWAS) of salt tolerance was performed using different models of analysis. Twenty-one QTLs were identified and two candidate genes named *OsSTL1* (*Oryza sativa* salt tolerance level 1) and *OsSTL2* (*Oryza sativa* salt tolerance level 2) were confirmed using sequence analysis. Haplotype and sequence analysis revealed that gene *OsSTL1* was a homolog of salt tolerance gene *SRP1* (Stress associated RNA-binding protein 1) in Arabidopsis. The hap1 of *OsSTL1* was identified as the superior haplotype and a non-synonymous SNP was most likely to be the functional site. We also determined that the level of salt tolerance was improved by combining haplotypes of different genes. Our study provides a foundation for molecular breeding and functional analysis of salt tolerance in rice seedlings.

## Introduction

Rice (*Oryza sativa* L.) is an important food crop^1^ and soil salinity is a major factor affecting its growth and productivity worldwide^2^. Approximately one-third of the land area on which rice is grown is affected by salinity^3–5^. Rice seedlings are particularly sensitive to biotic and abiotic stresses^6^. High salinity impedes water and nutrient absorption from the soil. This inhibits seedling growth and ultimately decreases yield^7,8^. Therefore, development of salt-tolerant varieties in rice is a very critical target of rice breeding programs in coastal areas^9,10^.

An understanding of the genetic basis of salinity tolerance is required to facilitate development of varieties with salinity tolerance using marker-assisted selection^11^. Salt tolerance in rice is controlled by multiple genes. Many QTLs for salt tolerance at the seedling stage have been reported^2,3,5,6,12,13^. However, only two genes have been cloned, namely, *SKC1*, and *qSE3*^5,14^. These genes have been applied to rice breeding^6^, but the actual mechanism of salt tolerance remains unclear^15^. Therefore, identification of further QTLs/genes related to salt tolerance will help in characterizing unknown salt tolerance mechanisms and facilitate breeding applications.

Genome-wide association studies have been widely used to identify genetic variants affecting complex traits, either by comparative analysis or correlation analysis, and have identified many SNPs associated with target traits^13,16–18^. For example, thirty-three candidate genes in a protein interaction network were associated with salt tolerance in rice seedlings in a genome-wide association study (GWAS) using 295 accessions^13^. Because molecular marker-assisted selection (MAS) demands explicit genetic architecture of agronomic traits^19^ identification of new QTLs/genes related to salt tolerance in rice and confirmation of elite alleles is necessary for their utilization in modern molecular breeding.

In the present study, we performed a GWAS using 664 cultivated rice accessions from the 3,000 Rice Genome (3K-RG)^1^. Integrated gene annotation, genetic variation, homology and haplotype analysis were performed to identify candidate genes and possible causal polymorphisms for salt tolerance traits. Our results provide insight into the genetic architecture of salt tolerance and markers derived from the newly identified genes will be useful in improving salinity stress tolerance in rice seedlings.

## Materials and Methods

### Plant materials and experiments

The rice diversity panel comprising 664 cultivated rice accessions from the 3K Rice Genome (3K-RG)^1,20^ included 226 genotypes from the mini-core collection that were selected from an original core set of 4,310 accessions^21^, and 438 lines from the International Rice Molecular Breeding Network^22^. Both collections have accessions from 44 countries representing major rice-growing regions of the world **(**Table S1**)**. In order to analyze evolutionary aspects we studied an additional publicly available set of 446 wild rice accessions from a previous report^23^. The public transcriptomic data of IR29 was download from NCBI (https://www.ncbi.nlm.nih.gov/gds), series accession ID: GSE119720.

### Phenotypic data for salt tolerance

After treatment with salt solution, the symptoms of salt damage in the roots and leaves of seedlings were assessed visually^24^. From July 5 to September 10 in 2015, the diversity panel was cultivated in hydroponic culture with open-air environment that the average temperature from 14 °C to 32 °C at Xinjiang academy of agricultural sciences with 0.9% salt solution for two months for trait identification. First, the rice seeds were surface-sterilized and soaked in water to promote germination for 2 days at 30 °C. For each variety, seven seeds with normal budding and the same growth potential were transplanted to a gauze-based foam board (30 cm × 42 cm). Each foam board containing 10 varieties was placed in a plastic box containing a 0.9% salt solution. The salt solution was replaced once every two weeks for about 2 months (Fig. S1**)**. Phenotyping was based on the International Rice Research Institute (IRRI) salt tolerance/alkali identification criteria (i.e., salt tolerance/alkali standard growth score and salt/alkali standard dead leaf percentage)^25–27^. After salt stress, the symptoms of plant leaves and roots were observed and recorded on the basis of seven criteria (Shoot length (SL), Root length (RL), Shoot fresh weight (SFW), Shoot dry weight (SDW), Root fresh weight (RFW), Root dry weight (RDW) and Salt tolerance level (STL)). Salt tolerance level (STL) was based on the percentage of dead leaf blade tissue.

### Genotypic data of 664 materials and GWAS for salt tolerance

Genetic variation (single nucleotide polymorphism (SNP)) data for the 664 accessions were obtained from the 3K-RG publicly available database, which included approximately 17 million highly credible SNPs and 2.4 million indels aligned to the cv. Nipponbare IRGSP 1.0 reference genome. Publicly available SNP set for the 446 wild rice accessions were downloaded from http://www.ncgr.ac.cn/RiceHap3^23^.

We identified 3,513,863, 2,280,242 and 1,855,669 SNPs as a credible SNP set after removing SNPs with missing rates >30% and minor allele frequencies <5% in full, *indica* and *japonica* populations. Principal component (PC) and kinship matrix analyses were performed in order to account for population structure. A neighbor-joining tree was constructed using 68,376 SNPs evenly distributed throughout the genome. The first three PC were used to construct the PC matrix. We performed a GWAS of salt tolerance traits of the 664 accessions using a compressed mixed linear model (CMLM) and general linear model (GLM) using the SNP set and default settings in GAPIT software. We also performed FaST-LMM using FaST-LMM software^28^ in order to compare three different models. Due to the non-independence of SNPs caused by strong LD, the thresholds derived from the total number of markers were usually too rigorous for detection of significant associations. Suggestive thresholds were calculated using the formula “-log10(1 / effective number of independent SNPs)” as described previously^28–30^ and effective numbers of independent SNPs were determined by PLINK^31^ (window size 50, step size 50, r^2^ ≥ 0.2) to be 117,880, 94,282 and 42,831 in the full population as well as in *indica* and *japonica* subpopulations, respectively. We divided the STL (Y) of each accession into the original genotypic effect (G) and the fixed effect of population structure (P). Through regression analysis of each PC on Y, we calculated the average effect of each PC in the PC matrix on each individual (P); G was excluding P from Y. Then G was randomly reshuffled as G-new, and the P + G-new was reconstructed to the novel phenotype of each accession. The conditional permutation test of total of 1,000 sets of P + Gr was performed using GLM with the same parameters and PC matrix. The threshold was therefore set at -log (*P*) = 4 to identify significant associations. LD heatmaps surrounding peaks in the GWAS were constructed using “LD heatmaps” in the R package^32^.

### Data analysis

Differences in phenotypic values between alleles of each non-synonymous SNP were assessed by Student’s t-tests. The sequence alignment of each gene was determined using non-synonymous SNPs associated with STL, and differences in phenotypic value between haplotypes of each gene were calculated by one-way ANOVA or Student’s t-tests. Duncan’s multiple range tests were conducted to make comparisons if the results of the one-way ANOVA were significant (*P* < 0.01). Nucleotide diversity (π)^33,34^ and Tajima’s *D*^35^ were calculated using an in-house Perl script.

## Results

### Population structure and phenotypic variation in ST traits among 664 cultivated rice accessions

**Figure 1:**
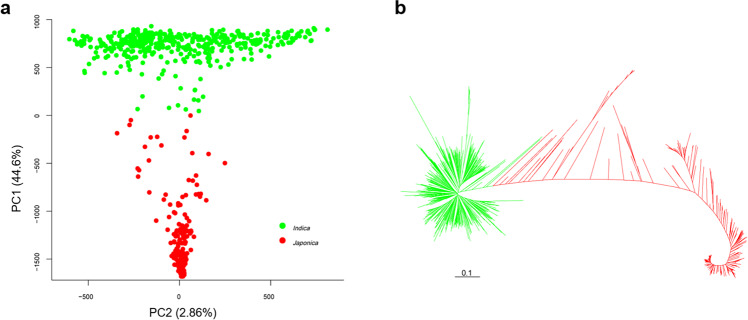
Population structure of 664 rice accessions. (**a**) Principal component analysis and (**b**) neighbor-joining tree for all accessions; green lines represent *indica*, and red lines represent *japonica*. PC analysis was performed using 3.5 million SNPs with missing data rates ≤ 30% and minor allele frequency ≥ 5%. Neighbor-joining tree was constructed using 68,376 SNPs evenly distributed throughout the genome.

Prior to GWAS we performed a PC and kinship matrix analysis based on 3.5 million SNPs to characterize the population structure (Fig. 1). There was a distinct subpopulation structure; PC1 separated the accessions into *indica* (428 accessions) and *japonica* (236 accessions) subpopulations explaining 44.6% of the total genetic variation. The kinship matrix and neighbor-joining tree also showed similar results (Figs. 1 and S2**)**.

In order to confirm differences in phenotype between the *indica* and *japonica* subpopulations we performed statistical analyses of all seven salt tolerance-related traits. There were significant differences between *indica* and *japonica* for RFW, RDW and SFW but no differences in RL, SL, SDW and STL **(**Fig. S3**)**. The descriptive statistics for the two sub-populations are provided in (Table 1) and the distribution for each trait is shown in Fig. S4. The mean STL were 4.91 and 5.14 in *indica* and *japonica*, respectively. These results indicated the presence of genetic variation in salt tolerance in both *indica* and *japonica*.

**Table 1:** Descriptive statistics for seven phenotypes in subpopulations.

Phenotype	Range		Mean ± SD		Skewness		Kurtosis		CV (%)	
	*Ind*	*Jap*	*Ind*	*Jap*	*Ind*	*Jap*	*Ind*	*Jap*	*Ind*	*Jap*
**SL (mm)**	**4.00~ 34.20**	**16.30~ 34.00**	**23.11 ± 3.78**	**23.69 ± 3.39**	**0.12**	**0.40**	**1.57**	**0.03**	**0.16**	**0.14**
**SFW (g)**	**0.18~ 1.23**	**0.23~ 0.92**	**0.48 ± 0.14**	**0.45 ± 0.12**	**0.89**	**0.97**	**3.06**	**1.22**	**0.29**	**0.27**
**SDW (g)**	**0.03~ 0.28**	**0.02~ 0.25**	**0.15 ± 0.04**	**0.14 ± 0.03**	**0.14**	**0.22**	**0.26**	**0.57**	**0.27**	**0.21**
**RL (mm)**	**8.80~ 24.60**	**9.60~ 26.30**	**15.41 ± 2.85**	**15.13 ± 2.80**	**0.41**	**0.84**	**0.17**	**1.72**	**0.18**	**0.19**
**RFW (g)**	**0.15~ 1.28**	**0.18~ 1.05**	**0.49 ± 0.15**	**0.46 ± 0.14**	**0.92**	**0.91**	**2.20**	**1.20**	**0.31**	**0.30**
**RDW (g)**	**0.02~ 0.14**	**0.02~ 0.12**	**0.06 ± 0.02**	**0.06 ± 0.02**	**0.88**	**1.01**	**2.21**	**1.57**	**0.33**	**0.33**
**STL**	**1~ 9**	**1~ 9**	**4.91 ± 1.80**	**5.14 ± 1.46**	**0.48**	**0.33**	**0.03**	**0.77**	**0.37**	**0.28**

CV: coefficient of variation; *Ind*, *Indica*; *Jap*, *Japonica*.

Correlation analysis showed that STL was significantly correlated with most aboveground and below ground traits. RFW and RDW were highly correlated (0.872), whereas the STL was not significantly correlated with RL (0.002) **(**Table S2). The STL of most varieties was about 5 and a very small proportion (11 accessions) had a low STL indicative of high salt tolerance. We therefore adopted STL as a meaningful indicator of salt tolerance and focused on salt tolerance traits in subsequent analyses.

### Identification of QTLs for STL by GWAS

**Figure 2:**
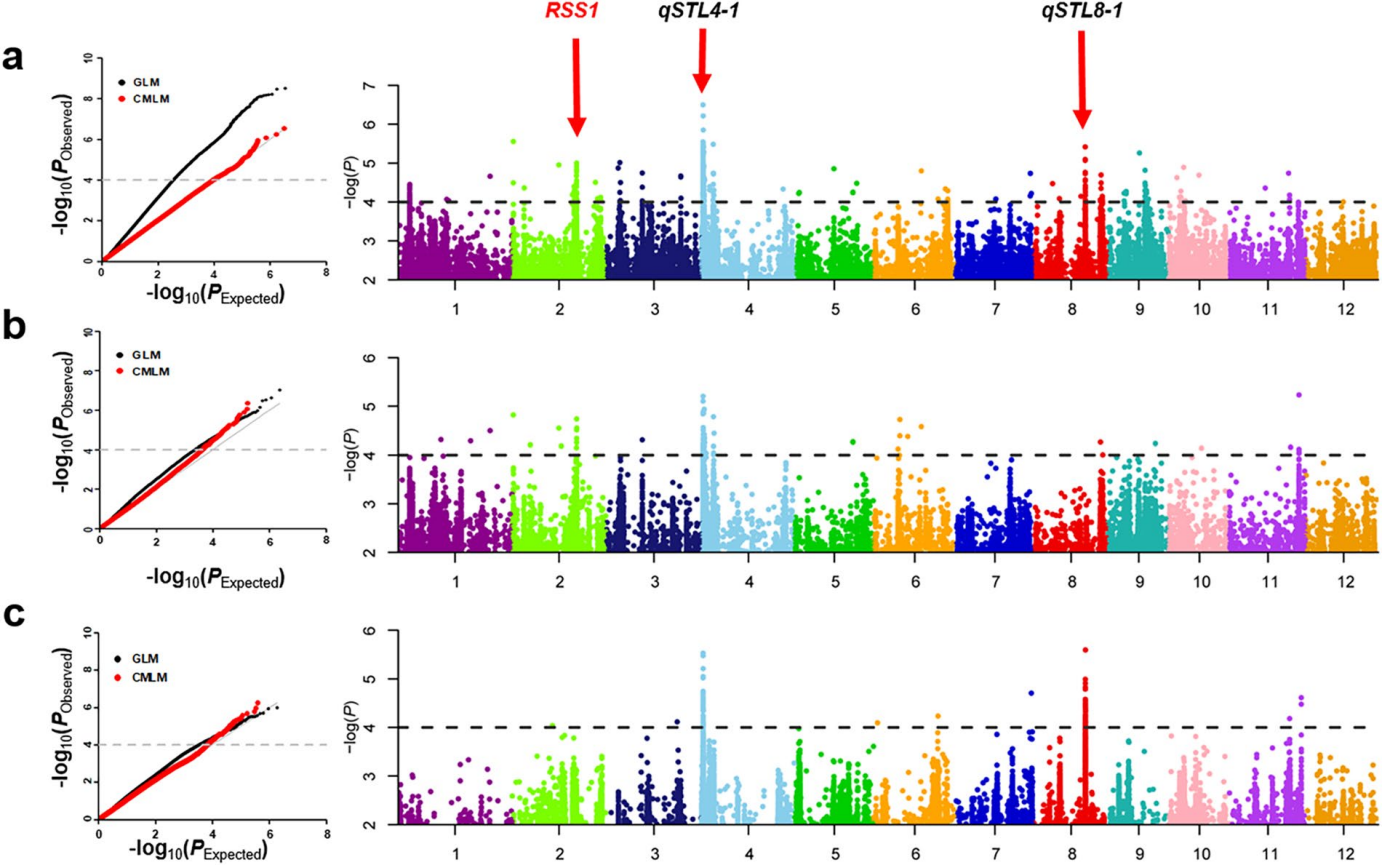
GWAS for salt tolerance in rice seedlings. Quantile–quantile plots and Manhattan plots for the GWAS in the full (**a**), *indica* (**b**) and *japonica* (**c**) populations using CMLM. In quantile-quantile plots, black dots are for GLM, and red points are for CMLM. In Manhattan plots, the gene in red was previously cloned, and QTLs in black were considered to be important. Dashed horizontal line for each population indicates the suggestive threshold (*P* = 1.0 × 10^−4^).

The general linear model (GLM), compressed mixed linear model (CMLM) and Factored Spectrally Transformed Linear Mixed Models (FaST-LMM) were performed using GAPIT and FaST-LMM to identify association signals in the *indica*, *japonica* and full populations, using the STL data and 3.5 million SNPs. Manhattan plots of STL show that detection using all three models was consist, especially CMLM and FaST-LMM (Figs. 2 and S5–6**)**.

**Figure 3:**
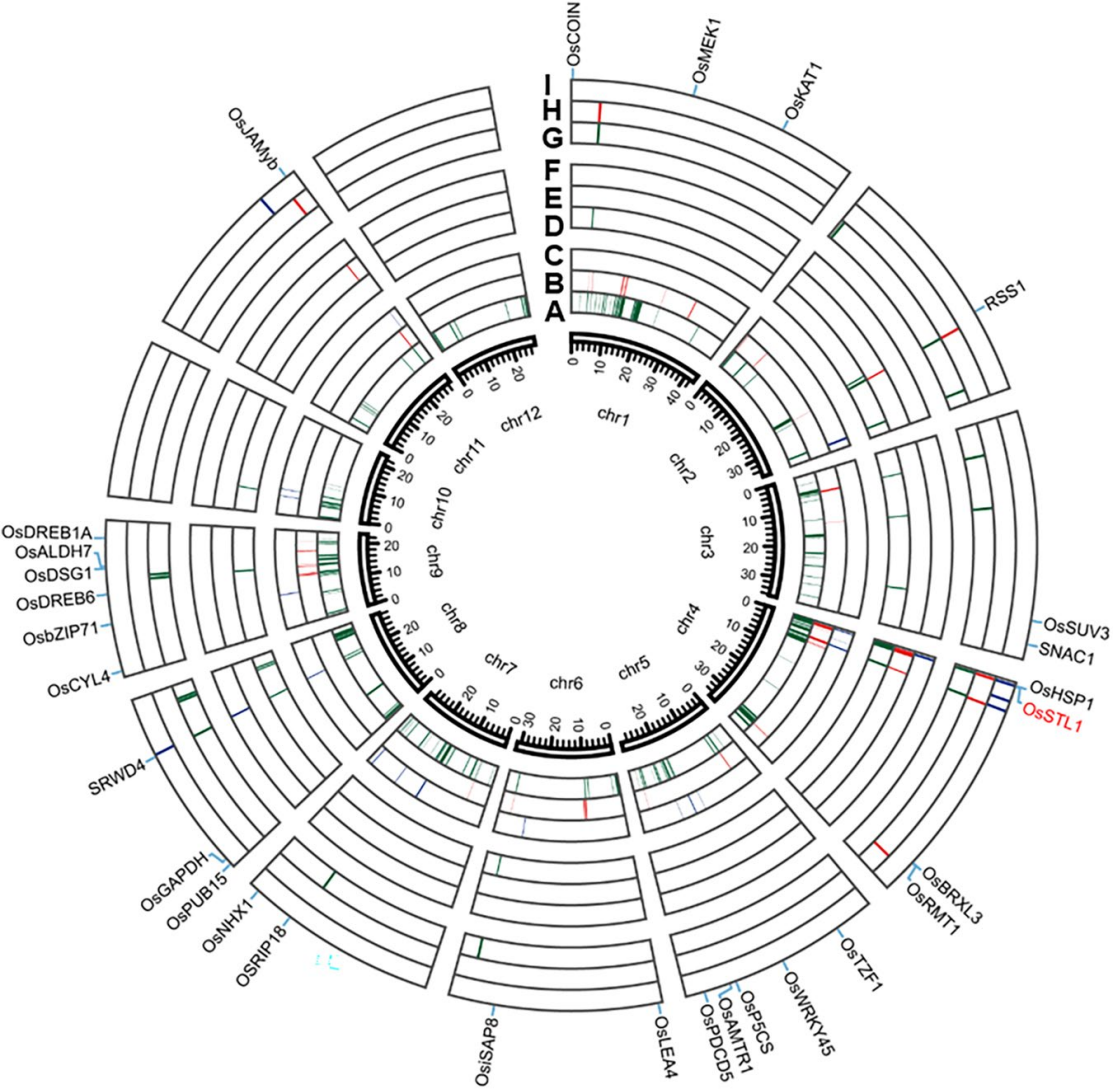
Circos map of all association signals for salt tolerance in the full population, *indica* and *japonica* using GLM, CMLM and FaST-LMM. The colored blocks in each layer from inner to outer represent QTLs detected from in the full population (**a**), *indica* subpopulation (**b**), *japonica* subpopulation (**c**) using GLM, and in the same populations (**d**,**e**,**f**) using CMLM and (**g**,**h**,**i**) using FaST-LMM, respectively. Twenty-eight known genes are labeled with black script at the outermost layer; red color represents candidate genes detected in this study.

We identified 321, 76, and 103 SNPs for STL by GWAS at -log (*P*) significance levels of 4 in the full population, *indica* and *japonica* subpopulations, respectively, when using CMLM (Fig. 2). With reported genome-wide linkage disequilibrium (LD) decay rates of up to 167 kb^16,23^ adjacent significant SNPs with distances less than 170 kb were merged into single association signals. The SNPs with the minimum *P* value in a signal region was considered the lead SNP. Fifteen, 4 and 2 QTLs were identified by GWAS using CMLM **(**Table S3), 124, 32 and 16 QTLs were identified using GLM model **(**Table S4**)**, and 15, 6 and 5 QTLs were identified using FaST-LMM **(**Table S5**)** in the full population, *indica* and *japonica*, respectively (Fig. 3). Most of the QTLs detected in CMLM (12/15, 4/4, 2/2) were also detected by FaST-LMM, and all QTLs identified by CMLM and FaST-LMM were included among those detected by GLM.

By minimizing false positives caused by population structure we determined that CMLM was the most suitable of the three models for detecting STL QTL in our populations by comparing the quantile-quantile plots from the three models in each population (Figs. 2 and S5–6). We also optimized the parameters in the CMLM for STL in each GWAS panel. Detailed comparisons identified *qSTL4-1* all three populations with Chr4_487087 as its lead SNP. Three of four QTLs in *indica* and both QTLs in *japonica* were also identified in the full population, indicating that these detected QTLs were stable in both sub-populations. The fact that almost half of the QTLs (10/21) overlapped with those found previously^36^ supported the reliability of the present results **(**Table S3**)**.

One of the QTLs detected by CMLM probably corresponded to the cloned salt tolerance gene *RSS1* (Fig. 3, Table S3**)**. The RSS1 protein has a putative role in maintenance and viability of meristematic cells in rice plants under salt stress^37^. There were as many as 28 and 1 cloned genes related to salt tolerance that corresponded to present QTLs detected by the GLM and FaST-LMM, respectively (Fig. 3, Tables S4-5**)**.

### Determination of candidate genes within selected QTLs

**Figure 4:**
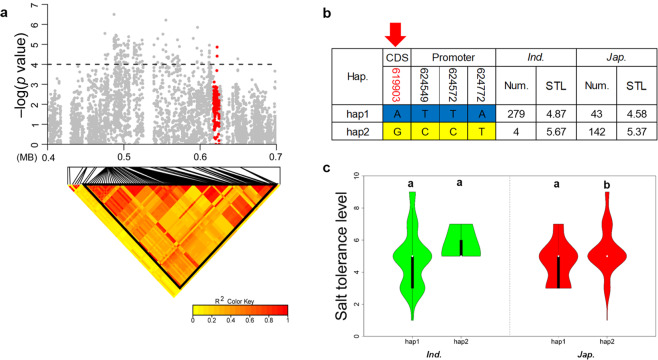
Dissection of *OsSTL1* for salt tolerance on chromosome 4. (**a**) Local Manhattan plot (upper) and LD heatmap (lower) surrounding the lead SNP for STL on chromosome 4. Red dots represent all SNPs within *OsSTL1*. (**b**) Different haplotypes of *OsSTL1* in *indica* and *japonica*. The red number identifies the nonsynonymous mutation; red arrow shows the functional site. (**c**) Comparison of the STL trait for salt tolerance among *OsSTL1* haplotypes in *indica* and *japonica* using one-way ANOVA. Green violins represent *indica* and red violins represent *japonica*. Different letters indicate significant differences (*p* < 0.01) detected by one-way ANOVA.

**Figure 5:**
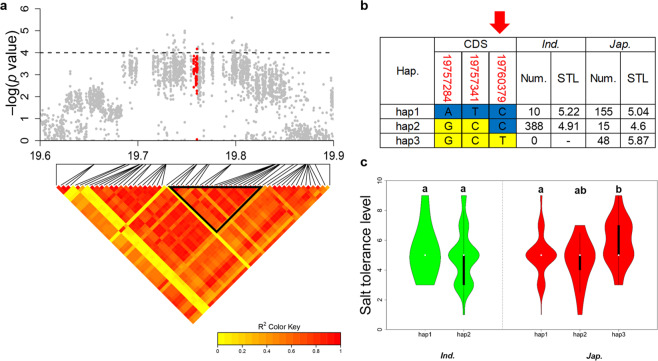
Dissection of *OsSTL2* for salt tolerance on chromosome 8. (**a**) Local Manhattan plot (upper) and LD heatmap (lower) surrounding the lead SNP for STL on chromosome 8. Red dots represent all SNPs within *OsOsSTL2*. (**b**) Different haplotypes of *OsSTL2* in *indica* and *japonica*. The red number identifies the nonsynonymous mutation; red arrow shows the functional site. (**c**) Comparison of the STL trait for salt tolerance among *OsSTL2* haplotypes in *indica* and *japonica* using one-way ANOVA. Green violins represent *indica* and red violins represent *japonica*. Different letters indicate significant differences (*p* < 0.01) detected by one-way ANOVA.

We chose two QTLs for detailed study; *qSTL8-1* was identified in the full population and *japonica* subpopulation, and was also identified in a previous report^36^ whereas *qSTL4-1* was detected in all three populations implying that they were worthy of pursuing to the candidate gene level (Figs. 4a and 5a**)**.

*qSTL4-1* contained 154 SNPs at suggestive thresholds of four. The candidate region of 0.49 Mb to 0.62 Mb in *qSTL4-1* was evaluated using pairwise LD correlations with a threshold r^2^ > 0.6^38^ (Fig. 4a). The candidate region in *qSTL4-1* contained 12 genes of which three were retrotransposons. The other 9 genes were possible candidate genes. Haplotype analysis was performed for each gene. One candidate gene had variable numbers of non-synonymous SNPs with a -log (*P*) value greater than two and significant SNPs within the promoter. There was no non-synonymous SNP with a -log (*P*) value greater than 2 in two genes (Fig. S7c & e) and the other 7 genes showed very significant differences between haplotypes in *indica* or *japonica* rice **(**Figs. 4, S7a-b,d,f-g**)**.

Two of the genes differing between *indica* and *japonica* were LOC_Os04g01780 and LOC_Os04g01920. According to the gene annotations LOC_Os04g01780 is an uncharacterized expressed ACR (Act Domain Repeat), COG1399 (Clusters of Orthologous Groups 1399) family gene, and LOC_Os04g01920 is an expressed protein gene. Many genes for salt tolerance have been annotated as zinc finger family members. The third gene in the group, LOC_Os04g02000, was annotated as a zinc finger family protein gene and therefore was more likely to be the candidate functional gene for salt tolerance. According to the public transcriptomic data (The public transcriptomic data of IR29 was download from NCBI (https://www.ncbi.nlm.nih.gov/gds), series accession ID: GSE119720), the RPKMR_Salt_/RPKM_Controls_ (RPKM, Reads Per Kilobase per Million mapped reads) of LOC_Os04g02000 expression in IR29 was greater than 1.4, indicating differential gene expression between salt and controls. This candidate gene was finally named as *OsSTL1*.

*qSTL8-1* contained total of 14 significant SNPs. The candidate region of 19.74 Mb to 19.85 Mb in qSTL8-1 was evaluated using pairwise LD correlations (at threshold r^2^ > 0.6) (Fig. 5a). Three genes were located in the candidate region and were therefore considered more likely to be candidate genes. Haplotype analysis was performed for each gene as above. LOC_Os08g31850 and LOC_Os08g31860 showed no significant difference between the *indica* and *japonica* subpopulations **(**Fig. S8a**)** whereas LOC_Os08g31870 was annotated as a cell division cycle protein gene, and showed significant differences within the *japonica* subpopulation, but not in *indica* (Figs. 5 and S8b**)**.

LOC_Os08g31870 is putatively expressed cell division cycle protein 48 and this led us to hypothesize that LOC_Os08g31870 was more likely to be the candidate functional gene. According to the public transcriptomic data (The public transcriptomic data of IR29 was download from NCBI (https://www.ncbi.nlm.nih.gov/gds), series accession ID: GSE119720), the RPKMR_Salt_/RPKM_Controls_ (RPKM, Reads Per Kilobase per Million mapped reads) of LOC_Os08g31870 expression in IR29 was greater than 2, indicating differential gene expression between salt and controls. This candidate gene was finally named as *OsSTL2*.

### Elite alleles and the origin of the *OsSTL1*

We focused on *OsSTL1* with one non-synonymous SNP in the coding region (Chr4_619903, base G-A, amino acid P-S) located on RanBP2-type domain and 3 SNPs in the promoter region; two haplotypes (hap1 and hap2) were identified (Fig. 4b). There was a significant difference in STL between hap1 and hap2 of *OsSTL1* in *japonica*; the mean STL of hap1 (4.58, 43 accessions) was lower than that for hap2 (5.37, 142 accessions). Therefore, hap1 of *OsSTL1* was a superior genotype and its frequency in *japonica* could be increased in that subpopulation in order to increase its overall salt tolerance (Fig. 4). On the other hand, this haplotype was almost fixed in *indica*. Comparing the two haplotypes, the non-synonymous mutation Chr4_619903 was more likely to be the functional site in *OsSTL1*.

We compared *OsSTL1* with homologs in other crops using BLAST and found that the homolog *SRP1* (AT2G17975) in Arabidopsis was involved in salt tolerance^39^. Stress associated RNA-binding protein 1 (*SRP1*) encodes a C2C2-type zinc finger protein in Arabidopsis. A knock-out mutation in the *srp1* allele reduced sensitivity to ABA and salt stress at all growth stages whereas *SRP1*-overexpressing seedlings were more sensitive to ABA and salt than wild type.

For confirmation of the superior haplotype and functional site of *OsSTL1* we investigated 446 wild rice (*O. rufipogon*) accessions using publicly available sequence data^23^ to determine the origin of *OsSTL1*. We compared the nucleotide diversity (π)^33,34^ and Tajima’s *D*^35^ values for wild rice with those for the *indica* and *japonica* subpopulations. The π value for *OsSTL1* increased from wild rice to *japonica*, π_*japonica*_/ π_wild_ > 2, D = 4.67, and deviated significantly from zero. It was possible that balanced selection occurred in *japonica* during its domestication **(**Table S6**)**. The π value for *OsSTL1* in *indica* was less than that for wild rice, π_wild_/π_*indica*_ > 2, D = −1.33 indicating positive selection in *indica*. Although the overall diversity in cultivated rice was slightly increased it was clear that *indica* and *japonica* had undergone different selection directions in adapting to different environments during domestication.

### Elite alleles and the origin of *OsSTL2*

We also evaluated *OsSTL2* in which there were three non-synonymous SNPs (Chr8_19757284, base A-G, amino acid L-P, Chr8_19757341, base T-C, amino acid H-R and Chr8_19760379, base C-T, amino acid E-K) and three haplotypes (hap1, hap2 and hap3) were identified (Fig. 5b). The performance of hap3 of *OsSTL2* differed significantly from hap1 and hap2 in *japonica*. There was no significant difference in STL between hap1 and hap2 of *OsSTL2* in both subpopulations. The hap1 and hap2 of *OsSTL2* was the superior genotype in both *indica* and *japonica* (Fig. 5). By comparing the two haplotypes, the SNP of Chr8_19760379 was most likely to the functional site. The identified superior genotype and functional site of the candidate gene will help to improve salinity tolerance in rice by breeding.

Nucleotide diversity (π) and Tajima’s *D* were calculated for *OsSTL2*. The four populations had the same level of diversity **(**Table S7**)** suggesting no past selection of *OsSTL2* in the different populations.

### Haplotype combinations of *OsSTL1* and *OsSTL2*

**Figure 6:**
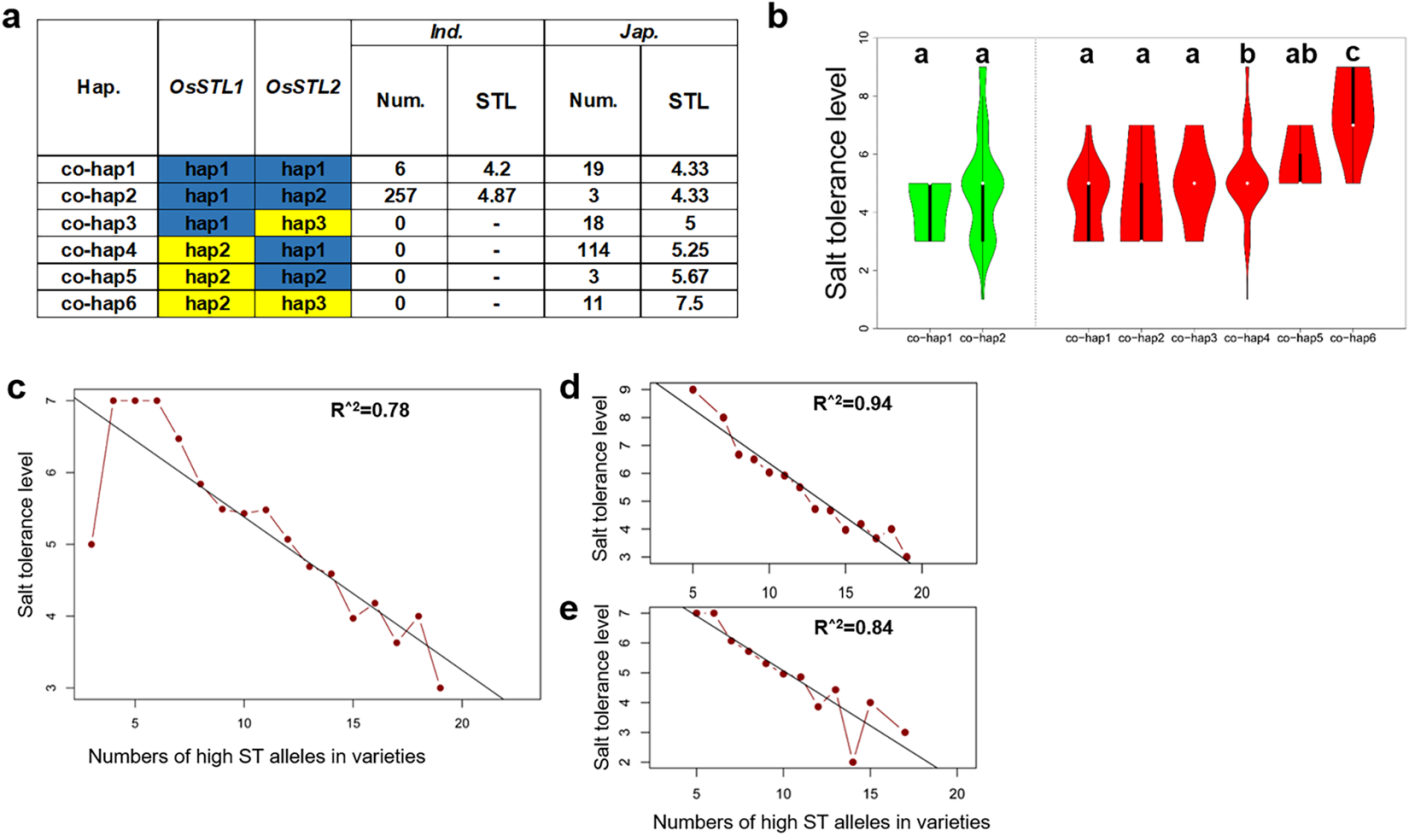
Functional validation of haplotype combinations of *OsSTL1* and *OsSTL2*. (**a**) STL for different haplotype combinations of *OsSTL1* and *OsSTL2*. Blue color represents superior alleles and yellow color represents inferior alleles. (**b**) Comparison of the STL trait for salt tolerance among haplotype combinations of *OsSTL1* and *OsSTL2* in *indica* and *japonica* using one-way ANOVA. Green violins represent *indica* and red violins represent *japonica*. Different letters indicate significant differences (*p* < 0.05) detected by one-way ANOVA. Pyramiding of lead SNP of high salt tolerance alleles in the full population (**c**), *indica* (**d**) and j*aponica* (**e**).

In order to improve breeding of salt tolerant genotypes for efficient utilization of *OsSTL1* and *OsSTL2*, we performed combination haplotype analysis of the two genes. The co-hap1 (combination with hap1 of *OsSTL1* and hap1 of *OsSTL2*) and co-hap2 (combination with hap1 of *OsSTL1* and hap2 of *OsSTL2*) represented the superior allele; the co-hap3(combination with hap1 of *OsSTL1* and hap3 of *OsSTL2*), co-hap4 (combination with hap2 of *OsSTL1* and hap1 of *OsSTL2*) and co-hap5 (combination with hap2 of *OsSTL1* and hap2 of *OsSTL2*) represented the intermediate allele, and co-hap6 (combination with hap2 of *OsSTL1* and hap3 of *OsSTL2*) represented the inferior allele. The mean STL of accessions with co-hap1 in *japonica* had significant lower values (p < 0.05) than those with co-hap4 and co-hap6, whereas, the mean STL of accessions with co-hap6 in *japonica* were significantly higher (p < 0.05) than those with other haplotypes as determined by one-way ANOVA (Fig. 6a,b). Based on these results, we concluded that the haplotypes of the two candidate genes were correctly identified and that pyramiding of the individually superior haplotypes improved the level of salt tolerance.

We screened 11 varieties with STL = 1 from the salt tolerance assays. Two were *japonica* and nine were *indica*. Ten of these varieties were co-hap2 **(**Table S8**)**. Clearly, the overall salinity tolerance level in breeding populations can be achieved by increasing the frequency of co-hap2.

## Discussion

In recent years, more than 170 genes for salt stress response have been cloned by forward or reverse genetic strategies in rice^5,14,15,40,41^. However, only a few reported genes for salt tolerance have been applied in rice breeding^5,6,14^. Gene pyramiding has become a powerful strategy to address complex traits^6^. Based on those studies, the salt tolerant genes will be used for new variety improvement by molecular assisted selection. However, molecular design breeding has not been made a great of progress because there have been no enough accurate genetic loci of the important agronomic traits and precise superior haplotypes dissected so far.

The resequencing based genotype provides vast natural variations, and the functional genes and elite alleles (superior haplotype) in natural populations could be explored by GWAS^13,42^. We detected the previously known salt stress tolerance gene *RSS1* in our QTL analysis using CMLM. Many previously salt stress response genes identified using mutants and gene differential expression with no mutations or few variations in our populations were not detected in this model. Therefore, different populations may be required to detect different functional genes.

Among 664 diverse rice accessions, we identified functional candidate genes *OsSTL1* and *OsSTL2*. We confirmed that hap1 of *OsSTL1* was superior haplotype, while hap1 and hap2 of *OsSTL2* were superior haplotypes. In addition, we identified the functional sites of the two candidate genes as the SNP Chr4_619903 and the Chr8_19760379, in which bases A and C caused the respective superior alleles.

The frequency of the A base allele in Chr4_619903 of *OsSTL1* in *japonica* was 23% (43 of 185 accessions), indicating that the utilization level of this allele was relatively low. Hence, the frequency of this allele can be increased to improve overall salt tolerance in *japonica*. By contrast the frequency of the same allele in *indica* was almost 98.5% and overall improvement in salt tolerance in this subspecies would require exploitation of the different genes. The frequency of the C base allele in Chr8_19760379 of *OsSTL2* was high in both *indica* (99.3%) and *japonica* (78.0%) indicating far less gains in overall salt tolerance being obtained by selection of alleles at this locus (Fig. 6a,b). These results suggested that *OsSTL1* in *indica* and *japonica* might have experienced different levels of selection in adaptation to different environments during domestication, however there was no evidence of differential selection of *OsSTL2* in the two subpopulations. Currently, the *OsSTL1* is most important genetic resource for molecular breeding to improve salt tolerance in *japonica*. The two candidate genes were only identified by GWAS and haplotype analysis, further functional tests will be needed.

Many major genes that significantly contribute to important agronomic traits can be pyramided to develop elite rice varieties^19,43^. The greatest gains are to be made by identification of the most superior functional allele (lead SNP) at each locus^38^. We identified the lead SNP in each of 21 QTLs and confirmed its superiority by phenotype. We then examined the correlation of the number of superior SNPs with STL values and found that STL increased with number of superior SNPs; the R^2^ values for the full population, *indica* and *japonic*a were 0.78, 0.94 and 0.84, respectively (Fig. 6c–e, Table S9**)**. These results indicate that as for many other traits, pyramiding of superior alleles of important genes for salinity stress will lead to new varieties with improved tolerance. Obviously the precise identification of superior haplotypes will have a critical bearing on the outcomes of molecular breeding.

## Supplementary information

Supplementary Figures.

Supplementary Table S1.

Supplementary Table S2.

Supplementary Table S3.

Supplementary Table S4.

Supplementary Table S5.

Supplementary Table S6.

Supplementary Table S7.

Supplementary Table S8.

Supplementary Table S9.
