# Supplementary material for: Genetic basis and identification of candidate genes for salt tolerance in rice by GWAS
Source: Sci Rep. 2020 Jun 19;10:9958. doi: 10.1038/s41598-020-66604-7 (PMC7305297; doi:10.1038/s41598-020-66604-7)
Supplement: Supplementary file 1 — Supplementary Figures. [file 41598_2020_66604_MOESM1_ESM.docx]

**Genetic basis and identification of candidate genes for salt tolerance in rice by GWAS**

Jie Yuan^1, 2#^, Xueqiang Wang^1#^, Yan Zhao^1#^, Najeeb Ullah Khan^1^, Zhiqiang Zhao^2^, Yanhong Zhang^2^, Xiaorong Wen^3^, Fusen, Tang^3^, Fengbin Wang^2*^ & Zichao Li^1*^

^1^ State Key Laboratory of Agrobiotechnology / Beijing Key Laboratory of Crop Genetic Improvement, College of Agronomy and Biotechnology, China Agricultural University, Beijing 100193, China;

^2^ Institute of Nuclear and Biological Technologies, Xinjiang Academy of Agricultural Sciences, Urumqi 830091, China;

^3^ Rice Experiment Stations in WenSu, Xinjiang Academy of Agricultural Sciences, Aksu 843000, China

# These authors contributed equally to this work.

* Corresponding author: Fengbin Wang (xjnkywfb@163.com) & Zichao Li ([lizichao@cau.edu.cn](mailto:lizichao@cau.edu.cn))

**Fig. S1** Hydroponic culture experiment using 0.9% NaCl for phenotyping salt tolerance of 664 cultivated rice varieties.

**Fig. S2** Kinship analyses of 664 rice accessions.

**Fig. S3** Comparisons of salt tolerance phenotypes within different groups; the X-axis indicates groups, and the Y-axis indicates traits. Different letters indicate significant differences (*p* <0.01) detected by one-way ANOVA.

**Fig. S4** Histograms of salt tolerance phenotypes between different groups: Full population **(a)**, *indica* **(b)** and *japonica* **(c)**; X-axis indicates different groups, and the Y-axis indicates the different traits.

**Fig. S5** GWAS for salt tolerance in rice seedlings. Manhattan plots for the GWAS in the full **(a)**, *indica* **(b)** and *japonica* **(c)** populations using GLM.

**Fig. S6** GWAS for salt tolerance in rice seedlings. Manhattan plot and quantile-quantile plots for the GWAS in the full (a), *indica* (b) and *japonica* (c) populations using FaST-LMM.

**Fig. S7** Haplotypes of candidate genes in *qSTL4-1* in *indica* and *japonica*. Gene structures and haplotype plots of **(a)** LOC_Os04g01750, **(b)** LOC_Os04g01780, **(c)** LOC_Os04g01800, **(d)** LOC_Os04g01810, **(e)** LOC_Os04g01874, **(f)** LOC_Os04g01920 and **(g)** LOC_Os04g01980. Red numbers show nonsynonymous mutations of each gene. Green violins represent *indica* and red violins represent *japonica.* Different letters indicate significant differences (*p* <0.01) detected by one-way ANOVA.

**Fig. S8** Haplotypes of candidate genes in *qSTL8-1* in *indica* and *japonica*. Gene structures and haplotype plots of **(a)** LOC_Os08g31850 and **(b)** LOC_Os08g31860. The red numbers show nonsynonymous mutations in each gene. Green violins represent *indica* and red violins represent *japonica.* Different letters indicate significant differences (*p* <0.01) detected by one-way ANOVA.
